# New Syphilis Cases in Older Adults, 2004–2019: An Analysis of Surveillance Data From South China

**DOI:** 10.3389/fmed.2021.781759

**Published:** 2021-12-02

**Authors:** Cheng Wang, Peizhen Zhao, Mingzhou Xiong, Joseph D. Tucker, Jason J. Ong, Brian J. Hall, Mojgan Sami, Heping Zheng, Bin Yang

**Affiliations:** ^1^Dermatology Hospital of Southern Medical University, Guangzhou, China; ^2^Guangdong Center for Skin Diseases and Sexually Transmitted Infections (STI) Control, Guangzhou, China; ^3^Sexually Transmitted Diseases (STD) Control Department, Southern Medical University Institute for Global Health and Sexually Transmitted Diseases, Guangzhou, China; ^4^University of North Carolina Project-China, Guangzhou, Guangdong, China; ^5^Faculty of Infectious and Tropical Diseases, London School of Hygiene and Tropical Medicine, London, United Kingdom; ^6^School of Medicine, Institute for Global Health and Infectious Diseases, University of North Carolina at Chapel Hill, Chapel Hill, NC, United States; ^7^Central Clinical School, Monash University, Melbourne, VIC, Australia; ^8^Department of Global Public Health, New York University Shanghai, Shanghai, China; ^9^Department of Health, Behavior, and Society, Johns Hopkins Bloomberg School of Public Health, Baltimore, MD, United States; ^10^Department of Public Health, California State University, Fullerton, Fullerton, CA, United States

**Keywords:** syphilis, older adults, surveillance, new diagnoses, determinants

## Abstract

**Background:** Sexual health among older adults is a major public health concern globally. The syphilis burden is increasing in older adults in China. This study aimed to describe factors associated with syphilis infection and diagnosis among older adults in China during a 16 year period.

**Methods:** Using 16 years of data (2004–2019) from the syphilis case report system of Guangdong, China, we compared data from older adults (aged ≥50 years) with those from younger people (aged 15–49 years). We compared the two age group with the Chi-square test for difference, and Joinpoint regression models to assess the temporal trends.

**Results:** During the study period, 242,115 new syphilis diagnoses were reported in older adults. The mean notification rate of new diagnoses was 64.1 per 100,000 population across the entire 16-year period, which significantly increased over time (average annual percent change [AAPC] 16.2%, 95% CI 13.7–18.7). Syphilis diagnoses increased significantly over time among less developed cities and older women. In 2019, compared with younger adults, newly diagnosed older adults were more likely to be male, native to reporting city, had unknown transmission routes, and were diagnosed late.

**Conclusion:** Our findings call for an urgent need to deliver more targeted prevention interventions for older adults, such as strengthen awareness among health care providers, and integration of syphilis services and primary health care for older adults.

## Background

The global population is rapidly ageing, with a predicted 22% increase in those aged 60 years or older from 800 million in 2011 to 2 billion people in 2050 (1). This presents a major global public health challenge in light of a growing burden of disease, which corresponds to a need for robust health care systems to adapt to evolving demands, and a call to invest in healthy ageing policies and programs throughout the world (2). Furthermore, by 2050, ~80% of older people will reside in middle- and low-income countries (LMIC), which deepens the crisis in regions with weak health systems (1).

Sexual health among the older adults is a critical concern (3) in light of their increasing rates of HIV and other sexually transmitted diseases (STIs), especially syphilis (4). Evidence from the United Kingdom (5), North America (4), Australia (6), and Korea (7) shows a significant rise of syphilis in the population group aged 50 years or older in recent years. Contributing factors may include limited knowledge of STIs, unsafe sexual practises, limited sexual health services targeting older adults, increased longevity and being widowed (4, 5). If not treated in a timely manner, syphilis can cause severe complications such as genital ulcers, chronic damage to the cardiovascular and nervous system, as well as increase the risk of acquiring and transmitting HIV (8). Studies suggest that older adults infected with STIs are more likely to present late and are at increased risk of getting these complications than younger adults (4, 9). However, increasing coverage and uptake of syphilis testing in older adults to promote early diagnosis and treatment is challenging for health care professionals (4). This may be even more difficult now due to the COVID-19 pandemic. Epidemiological research on syphilis is necessary to inform testing, treatment, and control of syphilis.

In the past decade, a rapidly increase of the relative portion of syphilis diagnoses reported among adults aged 50 years or older in China has been noted (10). Syphilis and other STIs were considered eliminated in China under the Maoist STI elimination campaign in the 1950s, but a resurgence was observed since the economic reform and opening up during the early 1980s (8). Estimating syphilis incidence is challenging since the infection might have occurred several years before symptoms arise or a diagnosis is made. A proxy is provided by surveillance of new syphilis cases reported over time. In settings like China with a rapidly ageing population (11) and sufficiently developed primary healthcare systems and disease reporting infrastructure (12), in-depth descriptive analyses of newly reported syphilis cases can inform policy decisions and the design of effective interventions for this age group. This can be particularly instructive for other LMIC settings undergoing similarly rapid social and economic change.

This study aims to describe factors associated with syphilis infection and diagnosis among adults aged 50 years or older by analysing new syphilis diagnoses reported in Guangdong Province, a high transmission region of southern China, using 16 years of data (2004–2019) from the provincial case report system.

## Methods

### Guangdong Province

Guangdong, China's richest Province, is an important global economic and trading hub in China. Guangdong is comprised of 21 municipalities with an apparent heterogeneity in economic development where the GDP per capita in the most developed municipality is eight times higher than the least developed municipality (13). Nine cities in the central area of Guangdong make up a more developed area known as the Pearl River Delta region. Similar to the epidemic trend in China, following decades-long STIs suppression from the 1950s to the 1970s under Maoist health policies, syphilis, like other STIs, has made a massive resurgence in Guangdong since the 1980s. Syphilis has been the second most commonly reported communicable disease in Guangdong Province for more than 15 years. Guangdong's prominence as the modern-day syphilis epicentre may be partially due to its highly developed STIs case reporting system, which has been collecting syphilis case reports since 1985. In China, all kinds of clinics and hospitals, including independent STI clinics, general hospital associated STI clinics, gynaecology and obstetrics clinics, family planning clinics, detention centres for sex workers and drug users and abortion clinics, are required by law to report newly diagnosed syphilis cases into the Chinese STD case report system. There are a total of 121 public STI clinics in Guangdong Province, with an average of 5.7 STI clinics in each of the 21 municipalities (13).

### Study Population and Data Sources

The study population included all individuals aged 50 years or older who were newly diagnosed with syphilis between Jan 1, 2004 and Dec 31, 2019. Confirmed syphilis cases were defined by two positive laboratory tests (non-treponemal test and treponemal test), history of sexual risk, and characteristic clinical manifestations (14). In accordance with previously published studies, we used 50 years of age as the cutoff to define older adults (3, 15). The primary study population was individuals aged 50 years or older at syphilis diagnosis, and the comparator group were individuals aged 15–49 years at the time of diagnosis. Case-level anonymised data were retrieved from the provincial case-based surveillance system maintained by the Guangdong Provincial Center for Skin Diseases and STI Control. Syphilis has been a reportable disease in Guangdong Province since 1985 and reported separately as primary, secondary, tertiary, congenital and latent syphilis cases since 1995. From 2004, a web-based case report system was established for syphilis case reporting. Case reports filled out by doctors who diagnose syphilis cases provide information on each case's demographic (gender, home location, marital status, occupation, and education), clinical (diagnostic evidence, onset of symptoms, stage of syphilis, presence of other STI), and epidemiological characteristics (route of acquisition). However, due to the high level of missing data (67–75%), the variables of occupation and education were not included in this analysis.

### Statistical Analysis

We analysed individuals who received a new, reported syphilis diagnosis including primary, secondary, tertiary, congenital and latent syphilis in 2019 by gender, transmission mode, migrant status, marriage, syphilis stage at diagnosis and co-infection with gonorrhoea and chlamydia. We compared the two age groups with the Chi-square test. Joinpoint regression models were used to estimate the average annual percentage change in the notification rate (AAPC) of new syphilis diagnosis with 95% CIs between 2004 and 2019, overall, by geography, gender and stages of syphilis. We defined statistical significance as a *p*-value of < 0.05. SAS version 9.4 (SAS Institute, Cary, NC) was used to analyse individuals' characteristics and compare differences. Joinpoint regression analysis was performed using the Joinpoint Regression Program version 4.9.0.0 (Statistical Research and Applications Branch, National Cancer Institute).

### Role of Funding Source

The funder of the study had no role in study design, data collection, data analysis, data interpretation, or writing of the report. The corresponding author had full access to all the data in the study and had final responsibility for the decision to submit for publication.

## Results

### Demographic Characteristics in 2019

**Figure 1:**
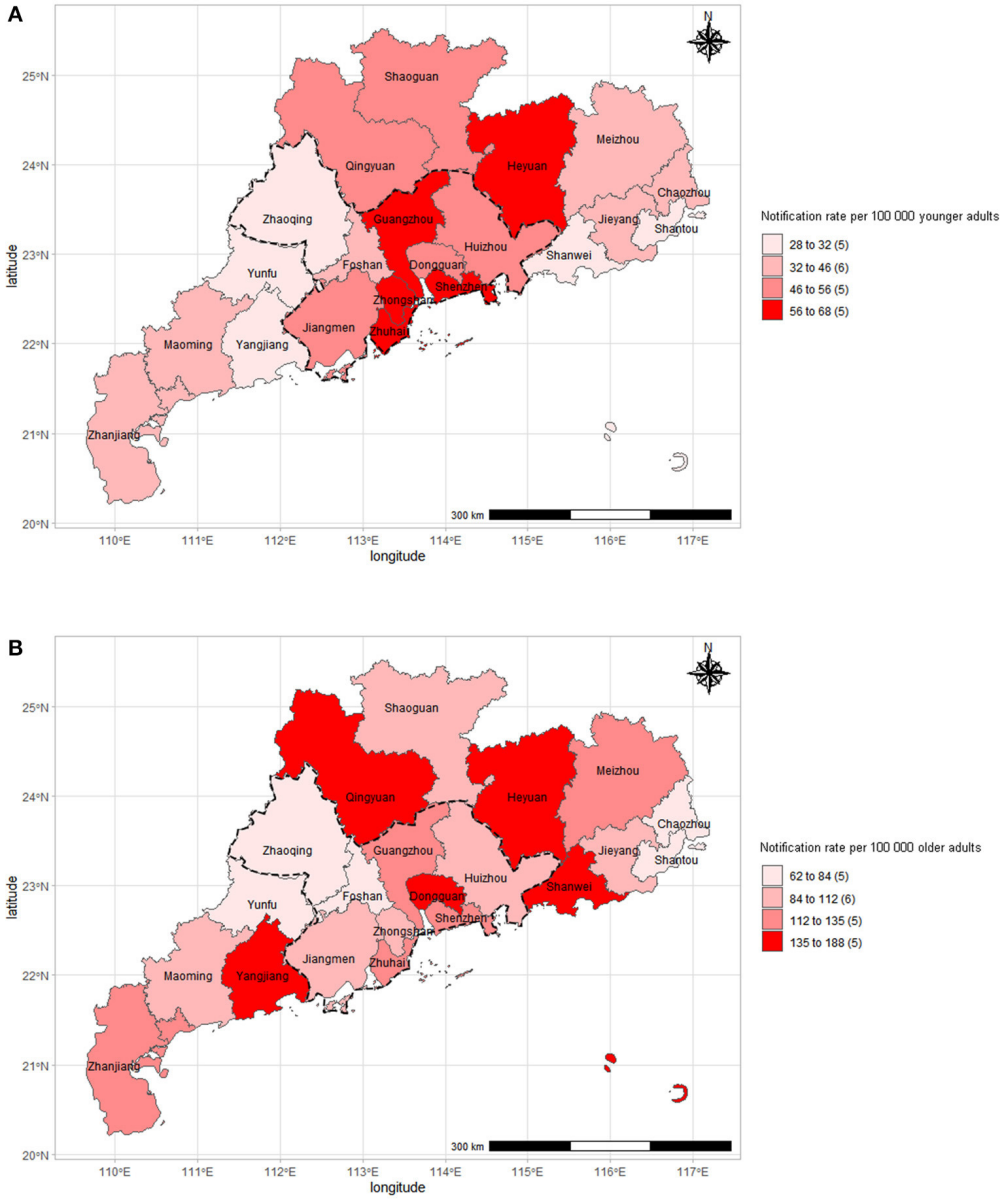
Geographic distribution of new syphilis diagnoses among older adults aged 50 years or older **(A)** and younger adults aged 15–49 **(B)** in Guangdong, China, 2019 (*N* = 71,055).

Of the 71 055 new syphilis diagnoses reported in 2019 in people aged 15 years or older in Guangdong, China, 34 699 (48.8%) were among older people (Table 1). The notification rate of new syphilis diagnoses among older people in 2019 was 112 per 100,000 population, ranging from 57.1 per 100,000 in Chaozhou (eastern Guangdong) to 142.9 per 100,000 in Heyuan (northern Guangdong) (Figure 1). By contrast, the notification rate of new syphilis diagnoses among younger people was 48.1 per 100,000 population in 2019, ranging from 22.5 per 100,000 in Yunfu (northern Guangdong) to 48.5 per 100,000 in Heyuan (northern Guangdong) (Figure 1).

**Table 1:** Characteristics of new syphilis diagnoses in people aged 15–49 years and those aged 50 years or older in Guangdong, China, 2019 (*N* = 71,055).

	**Aged 15–49 years (*n* = 36,356, 51.2%)**	**Aged ≥50 years (*n* = 34,699, 48.8%)**	**% Difference**	***P*-value**
Gender
Male	15699 (43.2)	22163 (63.9)	−20.7%	<0.0001
Female	20657 (56.8)	12536 (36.1)	+20.7%	
Transmission^*^
Gay	422 (1.2)	20 (0.1)	+1.1%	<0.0001
Heterosexual	7851 (21.6)	6140 (17.7)	+3.9%	
Injecting Drug use	98 (0.3)	24 (0.1)	+0.2%	
Unknown or Others	27985 (77.0)	28515 (82.2)	−5.2%	
Migration history
Native to reporting city	29445 (81.0)	30026 (86.5)	−5.5%	<0.0001
Other cities in Guangdong	2905 (8.0)	3043 (8.8)	−0.8%	
Other provinces	4006 (11.0)	1630 (4.7)	+6.3%	
Marriage^*^
Married	10754 (29.6)	13572 (39.1)	−9.5%	<0.0001
Unmarried	4414 (12.1)	652 (1.9)	+10.2%	
Divorced or widowed	472 (1.3)	1119 (3.2)	−1.9%	
Unknown	1752 (4.8)	1882 (5.4)	−0.6%	
Stage of syphilis
Primary	2359 (6.5)	1035 (3.0)	+3.5%	<0.0001
Secondary	2843 (7.8)	468 (1.3)	+6.5%	
Latent	30849 (84.9)	32947 (95.0)	−10.1	
Tertiary	136 (0.4)	248 (0.7)	−0.3%	
Congenital	169 (0.5)	1 (0.0)	+0.5%	
Co-infection (1/100,000)
Gonorrhoea	0.33	0.13	+0.23	<0.0001
Chlamydia	0.74	0.09	+0.46	

*Data are n(%) unless otherwise specified*.

* *Significance was computed after excluding data in the “unknown” categories*.

Among older adults diagnosed in 2019, men had a higher new syphilis diagnoses compared with women, with a male-to-female ratio of 1.8. The gender-specific notification rates of new diagnoses in 2019 were 144 per 100,000 older men and 80.4 per 100,000 older women. By contrast, among younger people, women had a higher new syphilis diagnoses compared to men, with a male-to-female ratio of 0.8 (Table 1).

Of cases diagnosed in 2019, a higher proportion of older adults vs. younger people were diagnosed late with latent syphilis and tertiary syphilis (*p* < 0.01). Reported transmission route (*p* < 0.01), migration history (*p* < 0.01), and marital status (*p* < 0.01) were also significantly associated with age, with older people being more likely to be classified as having an unknown or “other” route of transmission, native to the reporting city and married (Table 1).

In 2019, the notification rate of syphilis co-infection with gonorrhoea or chlamydia among older people was 0.13 per 100,000 population and 0.09 per 100,000 population, respectively, which were both significantly lower than among younger adults (*p* < 0.0001) (Table 1).

### Temporal and Geographic Trends on Syphilis From 2004 to 2019

**Figure 2:**
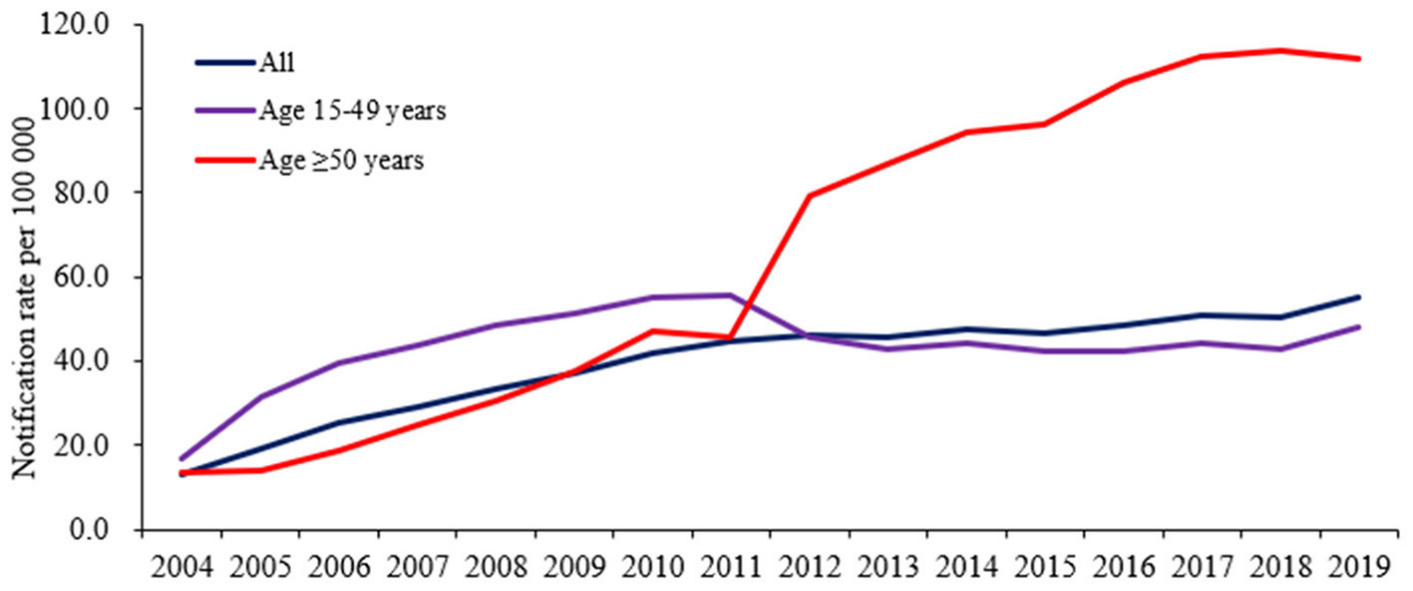
Annual notification rate of new syphilis diagnoses in Guangdong, China, 2004–2019.

Between 2004 and 2019, 242,115 new syphilis diagnoses were reported in older adults in Guangdong, China. The mean notification rate of new diagnoses was 64.1 per 100,000 population across the entire 16-year period, which significantly increased over time from 13.8 in 2004 to 112.0 in 2019 per 100,000 population (average annual percent change [AAPC] 16.2%, 95% CI 13.7–18.7). The average annual percent change during this period in the Pearl River Delta region, eastern, western and northern regions were 14.7% (95% CI 9.9–19.7), 23.7% (20.4–27.1), 20.6% (14.5–26.9), and 17.5% (13.6–21.5), respectively (Table 2; Figure 2).

**Table 2:** Change of annual notification rate of new syphilis diagnoses per 100,000 people in Guangdong, China, 2004–2019 (*N* = 641,665).

	**Aged** **≥50 years**			**Aged 15–49 years**		
	**ANR (2004–2019)**	**AAPC (95% CI)**	***P*-value**	**ANR (2004–2019)**	**AAPC (95% CI)**	***P*-value**
By geography
All cities	13.8–112.0	16.2 (13.7, 18.7)	<0.05	16.9–48.1	6.4 (2.6, 10.3)	<0.05
The pearl river delta area	17.2–109.0	14.7 (9.9, 19.7)	<0.05	25.0–53.2	4.0 (−1.8, 10.1)	<0.05
Eastern cities	8.0–98.8	23.7 (20.4, 27.1)	<0.05	3.7–36.7	16.3 (14.8, 17.8)	<0.05
Western cities	8.9–128.7	20.6 (14.5, 26.9)	<0.05	4.9–34.8	14.3 (10.4, 18.3)	<0.05
Northern cities	14.0–130.5	17.5 (13.6, 21.5)	<0.05	14.8–46.7	6.7 (5.1, 8.3)	<0.05
By gender
Male	20.0–144.0	15.0 (12.4, 17.6)	<0.05	14.9–39.8	5.9 (1.8, 10.1)	<0.05
Female	7.7–80.4	18.6 (15.8, 21.5)	<0.05	18.9–57.3	6.0 (3.1, 8.9)	<0.05
By stage
Primary	6.1–3.3	−3.4 (−7.5, 0.8)	<0.05	6.3–3.2	−7.5 (−10.8, −4.2)	<0.05
Secondary	4.9–1.6	−6.0 (−11.5, −0.1)	<0.05	6.0–4.0	−3.9 (−7.4, −0.3)	<0.05
Tertiary	0.2–0.8	9.6 (3.5, 16.0)	<0.05	0.0–0.2	-	-
Latent	2.6–106.3	28.4 (22.9, 34.1)	<0.05	4.5–40.8	14.4 (8.1, 21.1)	<0.05

*ANR, annual notification rate; AAPC, average annual percent change*.

Among younger adults, 399,550 cases were reported in the same period, with a mean notification rate of new syphilis diagnoses of 43.6 per 100,000 population across the entire 16-year period, which significantly increased over time as well from 16.9 in 2004 to 48.1 in 2019 per 100,000 (AAPC 6.4%, 95% CI 2.6–10.3). The average annual percent change during this period in the Pearl River Delta, eastern, western and northern regions was 4.0% (95% CI −1.8 to 10.1), 16.3% (95% CI 14.8–17.8), 14.3% (95% CI 10.4–18.3) and 6.7% (95% CI 5.1–8.3), respectively (Table 2; Figure 2).

### Gender Trends in Syphilis From 2004 to 2019

**Figure 3:**
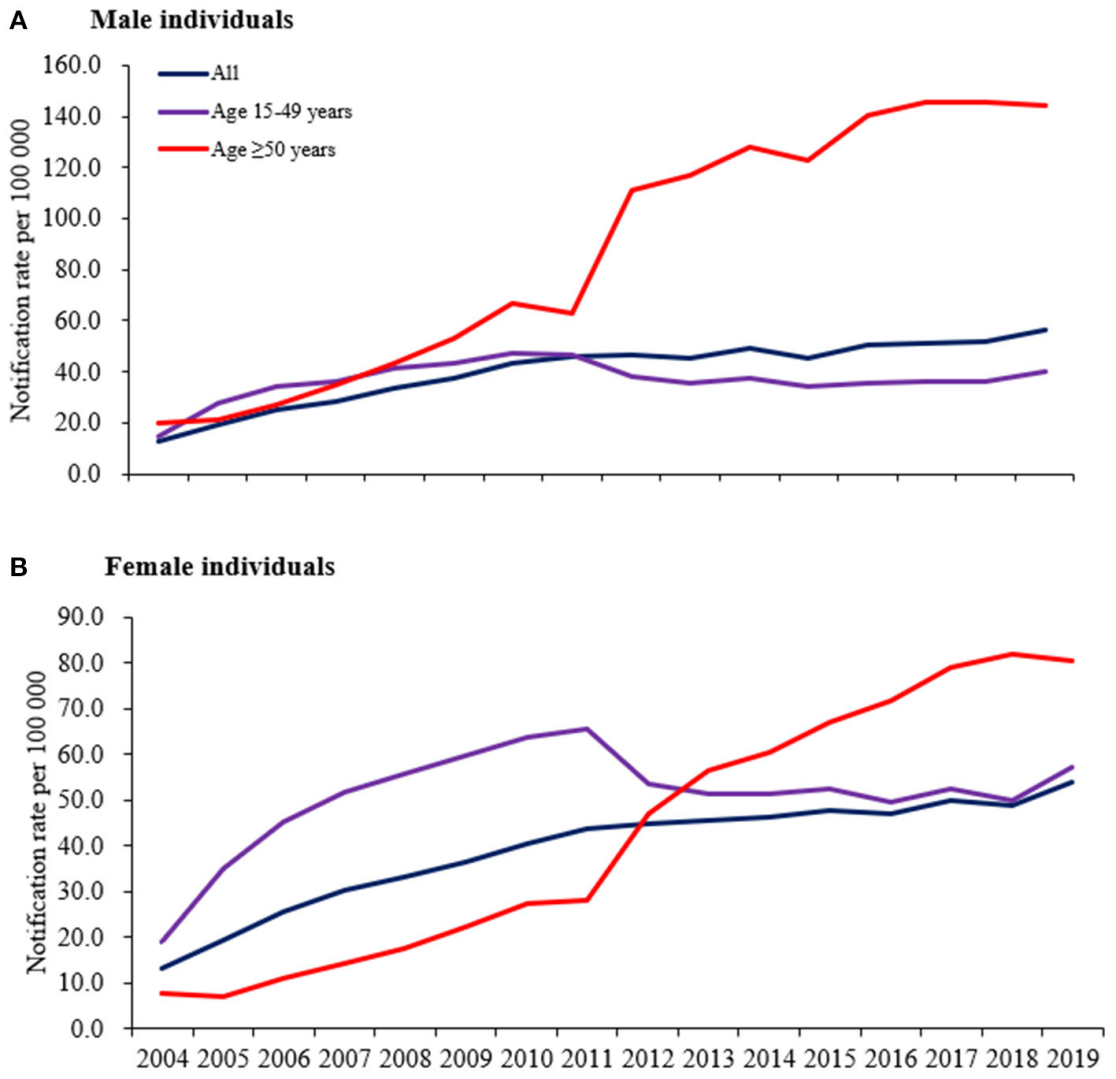
Annual notification rate of new syphilis diagnoses by male individuals **(A)** and female individuals **(B)** in Guangdong, China, 2004–2019.

An average annual notification rate of new diagnoses among older adults of 85.9 per 100,000 men and 42.0 per 100,000 women was found across the entire study period. The notification rate increased significantly from 2004 to 2019 in both sexes, from 20.0 to 144.0 per 100,000 men (AAPC 15.0%, 95% CI 12.4–17.6) and from 7.7 to 80.4 per 100,000 women (AAPC 18.6%, 95% CI 15.8–21.5). In younger people, the average annual notification rate of new diagnoses in men and women was 36.7 per 100,000 men and 51.1 per 100,000 women. A significant increase in new diagnoses in both sexes in this group was noted as well, from 14.9 to 39.8 per 100,000 men (AAPC 5.9%, 95% CI 1.8–10.1) and from 18.9 to 57.3 per 100,000 women (AAPC 6.0%, 95% CI 3.1–8.9) (Figure 3).

### Trends on Different Stages of Syphilis From 2004 to 2019

**Figure 4:**
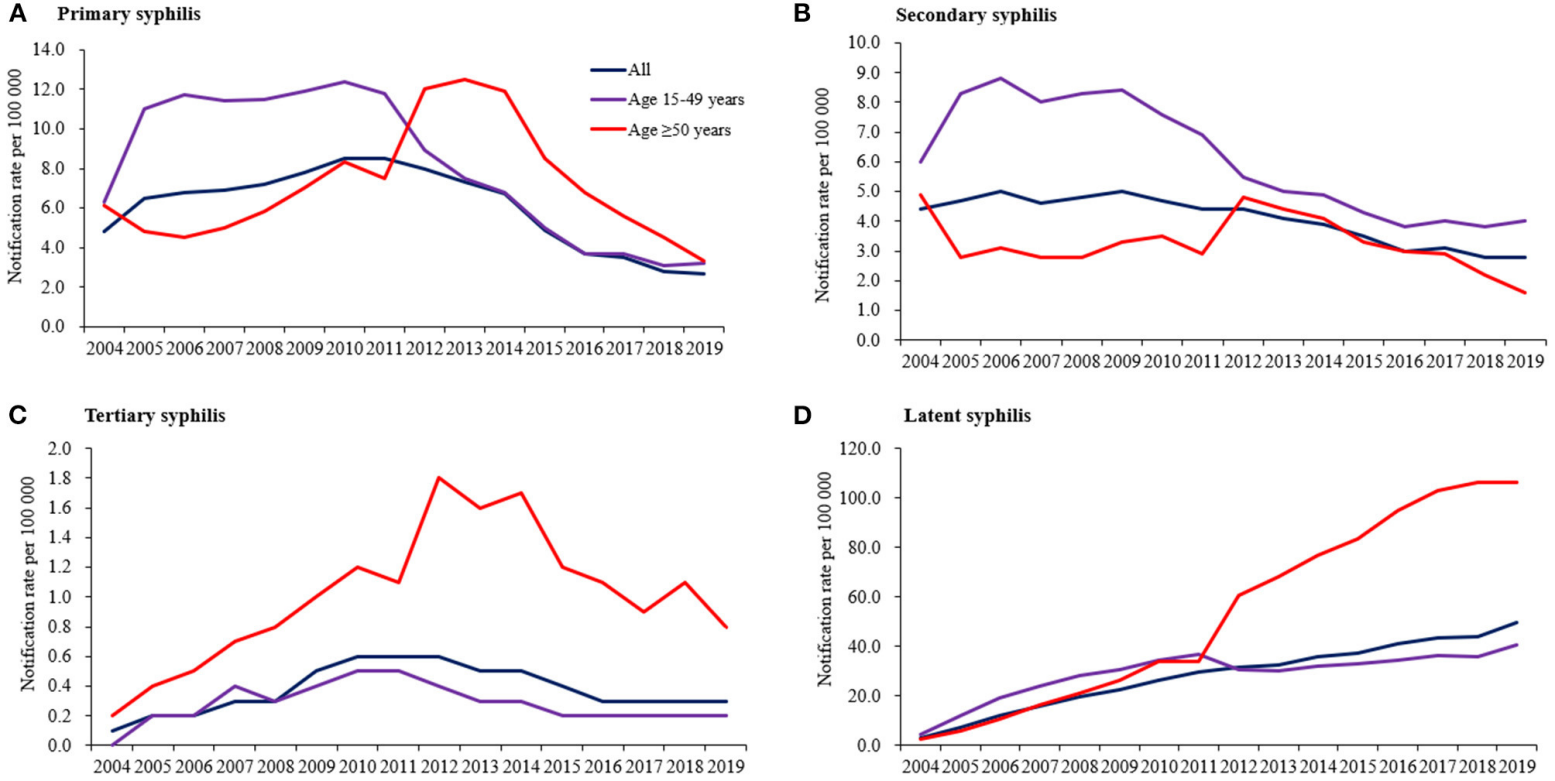
Annual notification rate of new syphilis diagnoses by primary syphilis **(A)**, secondary syphilis **(B)**, tertiary syphilis **(C)**, and latent syphilis **(D)** in Guangdong, China, 2004–2019.

In older adults, a significant increase in new latent syphilis diagnoses and tertiary syphilis diagnoses was observed, from 2.6 to 106.3 per 100,000 population (AAPC 28.4%, 95% CI 22.9–34.1) and from 0.2 to 0.8 per 100,000 population (AAPC 9.6%, 95% CI 3.5–16.0), respectively. A similar increasing trend in latent syphilis in younger people was noted as well but with a much lower AAPC, from 4.5 to 40.8 per 100,000 population (AAPC 14.4%, 95% CI 8.1–21.1). A significant decreasing trend was found in new primary syphilis diagnoses and secondary diagnoses in both age groups (Figure 4).

## Discussion

This study identified a notable increase in the notification rate of new syphilis diagnoses among older adults, which was much faster than in younger adults in Guangdong, China, over a 16-year period. This is consistent with findings of studies in another Chinese province (16), the United Kingdom (5), and the United States (17). A distinctive pattern of sociodemographic factors associated with an older adult newly diagnosed with syphilis was observed. Newly diagnosed older adults were more likely to be male, native to reporting city, had unknown transmission routes, and were diagnosed late compared with younger adults, consistent with several HIV studies in European countries (18, 19). However, there is a lack of available detailed epidemiology data on syphilis and other STIs in the older adults (4, 5). This study expands the literature by focusing on an LMIC context, including longitudinal data over a 16 year period, and exploring associated factors of syphilis infection and diagnosis.

Our findings showed that since 2012, the notification rate of new syphilis diagnoses in older adults was higher than that in younger adults. In 2011, the Chinese Ministry of Health launched a 10-year plan for syphilis prevention and control, including specific benchmarks for evaluating the success of the control program. This plan decreased the notification rate of new syphilis diagnoses in younger adults by 2020, but the same progress was not made for older adults. This might be attributable to more routine screening for latent syphilis in hospitals where older adults go because of their comorbidity such as hypertension, etc. Together with our findings, this might mirror an important issue globally, especially in LMIC where older people remain neglected by STI prevention, care and treatment services (4). Undoubtedly, the impact of the ongoing COVID-19 pandemic would make it more difficult for older adults to access those services (20). However, the issue of sexual health among older adults is just one facet of ageing-related problems (11). To address the sexual health problem, comprehensive measures from government authorities are needed, such as developing home and community care, promoting medical and care systems, integrating sexual health services into primary health care, strengthening active provider-initiated STI care services and ensuring an age-friendly environment (11).

By contrast with the trends among younger adults, we found a significant increasing trend in new tertiary syphilis diagnoses in older adults, which indicates a significant correlation between presenting with late syphilis diagnoses and older age. Late or tertiary syphilis can manifest years after infection as gummatous disease, cardiovascular disease, or central nervous system involvement (8). This is particularly concerning among older adults because it further increases the already higher morbidity in this age group than younger individuals. Various studies have shown that older people exhibit a longer delay period between symptom recognition and clinical presentation than younger people (15). Low perception of personal risk of STIs, limited knowledge on STIs and STIs transmission, and stigma associated with attending STI clinics might contribute to the late presentation in older adults (5, 21). These factors may also explain one of our finding that older adults were more likely to be classified as having an unknown transmission compared with younger adults, which is a common finding in Europe and the United states (15, 18, 19). However, even with timely clinical presentation, poor risk assessment, sexual history taking and advice on sexual health from healthcare providers may lead to late syphilis diagnoses in older adults (4, 22). Studies have indicated that, compared with younger individuals, newly diagnosed older people with STI were less likely to have been previously tested and were more likely to be diagnosed while in hospital for other, unrelated treatment (4). This might be due to the misconception of asexuality among older adults and that they are not at risk of getting STIs, which healthcare providers and society often share (23, 24). Developing more targeted sexual health promotion and STI prevention messages to older adults and strengthening active provider-initiated STI care services may help address this issue (4, 5).

We found that the city-specific burden of new syphilis diagnoses among older adults was heterogeneous, with cities in the northern region and western region generally reporting the highest syphilis notification rates in Guangdong, in contrast with the patterns among younger adults that the highest syphilis notification rates was generally reported by the cities in the Pearl River Delta region. This difference might be understood through the lens of Guangdong's labour migrant experience due to the vast territory and economic disequilibrium (12). The Pearl River Delta region is known as the national migration destination where the economic development is much higher than other regions in Guangdong. Nearly three-quarters of the Pearl River Delta population consists of migrants who tend to be young or of working age (11). By contrast, Guangdong's northern, western and southern region is mainly composed of older adults. This may contribute to our findings that the newly diagnosed older adults were more likely to be native to the reporting city compared to the younger adults (15). Additionally, our findings showed that men accounted for a larger proportion of syphilis cases than women in older adults. Demographers have shown that this “surplus group,” specifically “surplus men,” in the rural areas are mostly poor, uneducated and coming of age, which may have increased sexual risk and expending demand for commercial sex (25, 26). Together with the fact that limited STI healthcare services, including educational messages, routine screening, partner notification, and higher levels of stigma associated with health-seeking in rural areas (25), our findings calls for a new direction in which our efforts should be directed. However, to date, there have been very few studies of syphilis outside of urban China (26).

This study has implications for research and implementation. First, sexual health among older adults is a major public health concern but is often overlooked, especially in low- and middle- income countries. Our study expands the evidence base for the necessity of more programs and research focused on older adults' sexual health. Second, the increase in latent syphilis among older adults suggests the need for integration of syphilis services and primary health care in light of their comorbidity. One promising approach which could warrant scale-up is opt-out testing in primary health care (15).

This study has several limitations. First, all the data collected relied on mandatory reporting, which may be prone to information bias. Second, misclassification of syphilis stages could not be excluded, especially in places without sufficient training on syphilis diagnosis and case reporting. However, since 2011 when the national 10-year plan was launched, the Guangdong government has put considerable effort into training healthcare providers for mitigating these problems. Third, re-reporting of syphilis cases may exist. However, a survey of 235,215 syphilis cases reported between 2015 and 2018 in Guangdong showed that the duplicate reporting was 9.5%, which is lower than the requirement of 10% by Chinese authorities. Despite these challenges, our analysis provides important data from a large sample of people diagnosed with syphilis over 16 years.

In conclusion, our study provides data for the epidemiology of new syphilis diagnoses among older adults in South China. Increasing new syphilis diagnoses among older adults calls for an urgent need to deliver more targeted prevention interventions for this age group in addition to the total adult population, as well as strengthen awareness among healthcare providers.

## Data Availability Statement

The raw data supporting the conclusions of this article will be made available by the authors, without undue reservation.

## Author Contributions

CW developed the initial concept for the manuscript and drafted an initial manuscript. PZ and MX conducted the statistical analysis. JT, JO, BH, MS, HZ, and BY edited and contributed content to the final draft. All authors have read and approved the final manuscript.

## Funding

This study has been funded by the Dermatology Hospital of Southern Medical University.

## Conflict of Interest

The authors declare that the research was conducted in the absence of any commercial or financial relationships that could be construed as a potential conflict of interest.

## Publisher's Note

All claims expressed in this article are solely those of the authors and do not necessarily represent those of their affiliated organizations, or those of the publisher, the editors and the reviewers. Any product that may be evaluated in this article, or claim that may be made by its manufacturer, is not guaranteed or endorsed by the publisher.
